# P-747. Surgical Site Infections Following Cesarean Sections Across a Large Safety Net Public Hospital System

**DOI:** 10.1093/ofid/ofae631.943

**Published:** 2025-01-29

**Authors:** Khaleda Akter, Megan A Folks, Temilola-Azeezat Bakare, Briana Episcopia, Mary Fornek, Marie Abdallah, John Quale

**Affiliations:** SUNY Downstate Medical Center, Brooklyn, New York; NYC Health + Hospitals/Kings County, Brooklyn, New York; NYC Health and Hospitals Kings County, Brooklyn, New York; NYC H+H/ Kings County, Garden City, New York; New York City Health and Hospital, Manhattan, New York; New York City Health and Hospital/Kings County, Brooklyn, New York; New York City Health and Hospital/Kings County, Brooklyn, New York

## Abstract

**Background:**

Surgical site infections (SSIs) following Cesarean section (CSEC) can be associated with serious adverse outcomes. Pre-operative and intra-operative protocols have been established to minimize the risk of developing SSIs.

**Figure:**
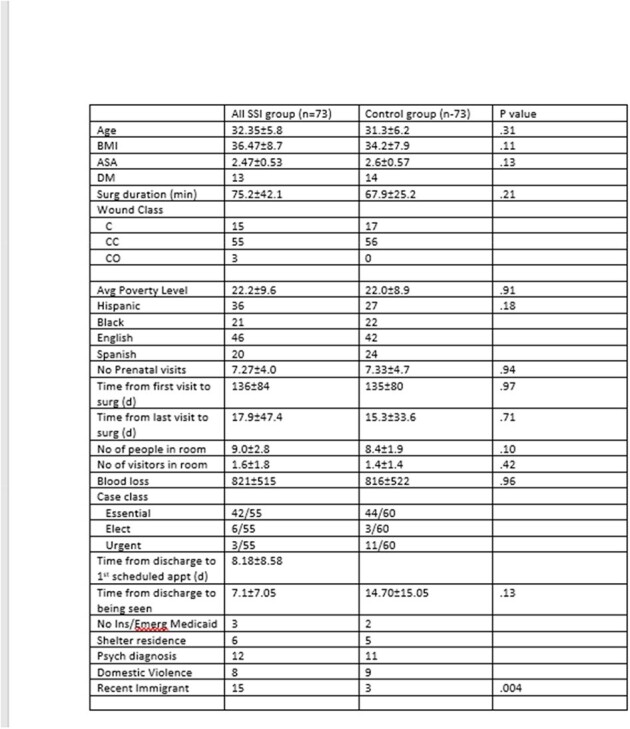

**Methods:**

Patients undergoing CSEC in an 11-hospital public health system in New York City in 2023 were reviewed. SSIs were defined according to National Healthcare Safety Network criteria. Propensity score matching, using six variables (age, body mass index, diabetes mellitus, surgery duration, American Society of Anesthesiology score, and wound class), was used to create a control group for the patients with SSIs.

**Results:**

Among the 4643 CSEC surgeries, 73 SSIs were recorded, including 49 superficial incisional, 6 deep incisional, and 18 deep/organ space SSIs. SSIs were detected during the initial admission in 7 cases, repeat hospitalizations in 33 cases, and in outpatient settings in 33 cases. A higher rate of SSIs was observed from four hospitals; zip codes for these patients had higher poverty levels than for those patients from the four hospitals with the lowest infection rates (23.4±8.6 vs. 17.7±9.7 per cent, P=.08).

Comparing the SSI and control groups, no differences were found in race, ethnicity, blood loss, or surgical team composition. Although both groups had a similar proportion of patients residing in shelters, the SSI group had more recent immigrants than the control group (15 vs. 3, P=.004). Notably, recent immigrants with SSIs had fewer prenatal visits compared to the rest of the SSI group (5.53±3.44 vs. 7.72±3.98, P=.04), and 14 of the 15 SSIs in the recent immigrants were detected post-discharge from CSEC.

**Conclusion:**

In a large public healthcare system, overall rates of SSIs following CSEC were low. Most infections were detected following discharge; living in neighborhoods of high poverty and being a recent immigrant appear to be risk factors for SSIs. While pre- and intra- operative protocols have been established to prevent SSIs, it appears postoperative factors (including wound care following discharge) warrant emphasis as well.

**Disclosures:**

**All Authors**: No reported disclosures

